# Measuring the Genuine Mismatch Negativity in the Auditory Multi‐Feature Paradigm

**DOI:** 10.1111/ejn.70362

**Published:** 2026-01-05

**Authors:** Andreas Widmann, Erich Schröger, Nicole Wetzel

**Affiliations:** ^1^ Wilhelm Wundt Institute for Psychology Leipzig University Leipzig Germany; ^2^ Leibniz Institute for Neurobiology Magdeburg Germany; ^3^ Center for Behavioral Brain Sciences Magdeburg Germany; ^4^ University of Applied Sciences Magdeburg‐Stendal Stendal Germany

**Keywords:** adaptation, auditory, genuine MMN, mismatch negativity, multi‐feature oddball paradigm, schizophrenia

## Abstract

The mismatch negativity (MMN) is a well‐studied event‐related potential (ERP) component in the EEG reflecting deviance detection in the auditory modality. It taps into the basic functioning of auditory regularity processing. The auditory multi‐feature paradigm is widely used in sensitive and special populations to measure the MMN simultaneously for different sound features in a short amount of time. It is consensus in the field that both adaptation and genuine deviance detection contribute to the “classic” MMN computed as deviant minus standard ERP difference. However, no attempts have yet been made to disentangle adaptation from the “genuine” MMN in the multi‐feature paradigm. Here, we propose a cascadic control condition for the auditory multi‐feature paradigm that controls for adaptation and physical differences between standard and deviant sounds. Using this new paradigm, we measured the genuine MMN, computed as deviant minus control ERP difference, for frequency, location, intensity, and duration deviants. The genuine MMN amplitudes for frequency and location were found substantially smaller than in traditional paradigms. No genuine intensity MMN and only a later and smaller genuine duration MMN were found. The results suggest stronger contributions of adaptation than in the traditional oddball paradigm. Controlling for adaptation is particularly relevant in research concerning predictive processing and the use of the MMN as a biomarker related to impaired NMDA receptor synaptic transmission as observed in schizophrenia. The presented multi‐feature cascadic control condition enables the measurement of the genuine MMN, which presumably reflects higher‐order cortical computations, such as predictive processing, still in a short amount of time.

## Graphical abstract

We designed and evaluated a cascadic control condition for the auditory multi‐feature oddball paradigm to control for the contribution of physical differences and adaptation to the mismatch negativity (MMN). Smaller, later, or no “genuine” deviant minus control MMNs were found for four sound features compared to “classic” deviant minus standard MMNs. The application of the control condition appears feasible as it allows to separate higher‐order cognitive processes from adaptation in sensitive populations.

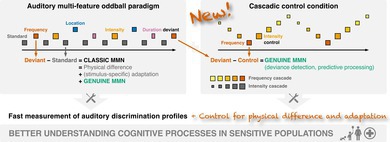

**Abbreviations**
ANOVA: analysis of variance
BF: Bayes factor
dB SPL: decibel sound pressure level
EEG: electroencephalogram
EOG: electrooculogram
ERP: event‐related potential
FIR: finite impulse response
ICA: independent component analysis
ITD: interaural time difference
LH: left hemisphere
M: mean
MEG: magnetoencephalogram
MMN: mismatch negativity
NMDA: N‐methyl‐D‐aspartate
OSF: Open Science Framework
PC: personal computer
RH: right hemisphere
ROI: region of interest
SD: standard deviation
SOA: stimulus‐onset asynchrony
TW: time window

## 1 Introduction

The mismatch negativity (MMN) component of the event‐related brain potentials (ERP), first described more than 40 years ago (e.g., Näätänen et al. 1978), is one of the best researched ERP components with contributions from a wide area of disciplines such as neuroscience, psychology, psychiatry, physiology, linguistics, and others. It is elicited in response to randomly occurring, rare, irregular sounds—deviants—different in some sound feature from a context of frequent, repeating, regular sounds—standards—in the so‐called oddball paradigm (in the following referred to as the “traditional” oddball paradigm). The MMN is elicited from deviations in various basic sound features such as pitch, location, or intensity (for review, see, e.g., Schröger 1998; Näätänen et al. 2019; Fitzgerald and Todd 2020) to increasingly complex and abstract deviants (for review, see, e.g., Paavilainen 2013) and also in other modalities, for example, in vision (for review, see, e.g., Stefanics et al. 2014; see, Male et al. 2020; Male 2025 for discussion). In adults, the auditory MMN typically has a peak latency between 100 and 250 ms after stimulus or deviation onset and a topography with a negative minimum over fronto‐central scalp sites inverting polarity at electrode sites below the Sylvian fissure (in the EEG with nose reference) typically measured with electrodes attached to the mastoids. The MMN is best visible as a negative deflection in the deviant minus standard ERP difference wave. Two influential extensions of the “traditional” oddball paradigm have been established: Schröger and Wolff (1996) suggested the inclusion of a so‐called “equiprobable” control condition accounting for the contribution of adaptation to the observed MMN component. Näätänen et al. (2004) suggested the so‐called “multi‐feature paradigm” greatly reducing experiment duration facilitating the measurement of the MMN in sensitive, for example, clinical or developmental populations. So far however, a combination of both these extensions is missing for the auditory modality. Therefore here, we shortly introduce both extensions and review important implications of the multi‐feature paradigm. We will adapt and test an experimental protocol (suggested previously by Male et al. 2020 for the visual modality) accounting for the contribution of adaptation to the MMN observed in the auditory multi‐feature paradigm.

### 1.1 The Contribution of Adaptation to the “Classic” MMN

We would consider it as consensus in the field that, in addition to the mismatch signal, the (stimulus‐specific) adaptation of neuronal assemblies by the repeated presentation of standard sound results in reduced ERP amplitudes (Nelken 2014). This at least contributes to the empirically observed MMN response when subtracting deviants and standards. In the following this is referred to as the “classic” MMN (Carbajal et al. 2024; Carbajal and Malmierca 2018; for review, see, e.g., Fishman and Steinschneider 2012). Two control conditions have been suggested to correct for the contribution of adaptation. The difference between deviant and control stimuli is commonly referred to as the “genuine” MMN (following Paavilainen et al. 1991). Although both adaptation and the genuine MMN reflect deviance‐sensitive processes, the genuine MMN is assumed to reflect higher order cortical computations such as genuine deviance detection, memory comparison (Näätänen et al. 2005), model updating (Winkler et al. 1996), or predictive processing (Winkler et al. 2009; Wacongne et al. 2012; Wacongne 2016). Predictive coding integrates both learning (optimization of model parameters; plasticity) and adaptation (optimization of precision or synaptic gain; efficacy) in a single formal theory (Garrido et al. 2009; Wacongne 2016) and has been mapped to different levels along the auditory pathway to explain auditory deviance detection (Carbajal and Malmierca 2018; Parras et al. 2021).

Schröger and Wolff (1996) suggested the so‐called equiprobable adaptation control condition (sometimes also referred to as “many‐standards” condition, e.g., Grimm et al. 2016). Several sounds systematically varying within the deviant feature are presented with deviant probability each in random order. As there is no regularity all control sounds can be considered as “standards” sharing the same presentation probability and therefore adaptational state as the deviant. One control sound is physically identical to the deviant. However, as receptive fields of standards and deviants may overlap and therefore standards may also adapt neuronal assemblies encoding deviants to some extent, it has been argued that adaptation is potentially overestimated in the equiprobable control. Furthermore, the equiprobable control provides a highly variable, unpredictable context in contrast to the regular context in the traditional oddball paradigm potentially affecting predictive processing. Ruhnau et al. (2012) suggested the cascadic control condition solving both problems. In contrast to the equiprobable condition, sounds are not presented in random order, but the relevant feature is systematically varied in ascending and descending order (e.g., F5–F4–F3–*F2*–**F1**–*F2*–F3–F4). A regular context allowing the generation of expectations on the upcoming stimulus is provided. The stimulus preceding the relevant control stimulus typically matches the traditional oddball standard, better equalizing the adaptational state of physically identical control and deviant stimuli.

### 1.2 The Multi‐Feature Auditory Oddball Paradigm

The MMN has also been extensively and successfully studied in sensitive, for example, clinical and developmental populations. One major problem in the application of MMN paradigms in sensitive populations is the duration of the experiments, in particular if several conditions or sound features should be tested. To solve this problem Näätänen et al. (2004) have suggested the auditory multi‐feature oddball paradigm, frequently also termed the “optimal” paradigm. The multi‐feature oddball paradigm intends to measure the MMN in response to several sound features concurrently. In the most commonly adapted “Optimum‐1” protocol (Näätänen et al. 2004), standard and deviant sounds are presented predictively in alternate trials (Table 1). Deviant sounds differ from standards in one of several sound features, for example, frequency, location, intensity, duration, or envelope (e.g., gap). The underlying rationale is that the deviants match the standard in all except one sound feature and thus “strengthen the memory trace of the standard with respect to those [the] levels of stimulus attributes they had in common” (Näätänen et al. 2004, 141; Pakarinen et al. 2007, 178). In other words, for example, in the case of four tested sound features, seven out of eight trials have invariant frequency, and one trial has a deviant sound frequency. Therefore, deviations in the frequency sound feature in the multi‐feature oddball paradigm have the same 12.5% probability as in the traditional oddball paradigm including 12.5% deviant trials. The MMNs in response to four or five deviant sound features can be recorded in the same time as for a single sound feature in the traditional oddball paradigm. Several modifications of the multi‐feature oddball paradigm have been suggested, for example, differing in (non‐exhaustive):

- the number of standard sounds between successive deviants (e.g., “Optimum‐2” with three consecutive standards, Näätänen et al. 2004; or the “no‐standard” paradigm omitting any standards, Pakarinen et al. 2010)
- the number of deviant sound features tested concurrently (e.g., “Optimal‐3” with three sound features, Fisher et al. 2011)
- the number of magnitudes of deviation per feature (e.g., to generate discrimination profiles, Pakarinen et al. 2007)
- stimulus material (e.g., musical stimuli, Vuust et al. 2011; language stimuli, Pakarinen et al. 2009)

**TABLE 1:** Illustration of the auditory multi‐feature oddball paradigm. In a block of eight trials, every second sound is a standard sound (here, Trials 1, 3, 5, and 7) characterized by four constant features (level 2; marked in italics): frequency (F2), location (L2), intensity (I2), and duration (D2). Every other trial is a deviant sound (shaded in gray). Deviant sounds pseudo‐randomly differ in one feature (marked in bold), from the standard *either* in frequency (F1, Trial 2), location (L1, Trial 4), intensity (I1, Trial 6), or duration (D1, Trial 8). The standard sound features are presented in seven out of eight trials (87.5%, and “strengthen the memory trace of the standard,” Näätänen et al. 2004), the deviant sound feature in one out of eight trials (12.5%) as in the traditional oddball paradigm.

Feature level	Trial 1	Trial 2	Trial 3	Trial 4	Trial 5	Trial 6	Trial 7	Trial 8
Level 2 (standard)	*F2 L2 I2 D2*	*L2 I2 D2*	*F2 L2 I2 D2*	*F2 I2 D2*	*F2 L2 I2 D2*	*F2 L2 D2*	*F2 L2 I2 D2*	*F2 L2 I2*
Level 1 (deviant)		**F1**		**L1**		**I1**		**D1**

Näätänen et al. (2004) asserted that the MMN can be measured in the multi‐feature oddball paradigm “without compromising the MMN amplitude” (Näätänen et al. 2004, 143).

The multi‐feature oddball paradigm has been widely adopted with a strong focus on research with sensitive and special populations in the field of hearing (e.g., cochlear implants, hearing aids, and tinnitus), mental (e.g., schizophrenia, depression, and post‐traumatic stress disorder), or developmental disorders (e.g., dyslexia and autism spectrum disorder), cognitive functions (e.g., attention, speech, and language), pharmacology, music, development, and others. There are, however, several implicit assumptions underlying the multi‐feature oddball paradigm as it is commonly applied, whose validity has not yet been empirically tested:

1. *Transition probabilities and context* differ from the traditional oddball paradigm. The multi‐feature oddball paradigm (implicitly) presumes that transition probabilities and context do not affect elicitation of the MMN. In the traditional oddball, the transition probability of a deviant following a standard approximately equals the probability of deviants (for discussion, see Schröger et al. 2023), and the deviant feature is fully predictable. In the multi‐feature oddball, the transition probability of a deviant following a standard is one (fully predictable), whereas the predictability of the deviant feature is one divided by the number of sound features tested. The context in the traditional oddball paradigm is regular (two sounds, mostly standards), whereas the context of the multi‐feature oddball paradigm is considerably more variable (number of tested sound features plus one sound, 50% standards).
2. The multi‐feature oddball paradigm presumes that *sound features are processed independently*. It has been shown that the amplitude of the MMN elicited by double (or triple) deviants violating several sound features simultaneously may be smaller than the sum of the MMN amplitudes elicited by the corresponding single feature deviants for certain feature combinations (Paavilainen et al. 2001, 2003; Wolff and Schröger 2001). That is, the stimulus representation underlying the MMN is at least partly integrated rather than feature specific (for a deeper discussion of separated vs. integrated feature representation, also see Näätänen and Winkler 1999). Also, for language‐related stimuli, it has been demonstrated that features may be processed in an integrated rather than separated manner (e.g., Gao et al. 2012; Lidji et al. 2009, 2010; we do not explore language‐related stimuli in this study).
3. *Comparison of physically identical* vs. *different stimuli* for standards and deviants. With some exceptions (e.g., Althen et al. 2016; Leung et al. 2012; Pakarinen et al. 2009 for sound duration), most studies applying the multi‐feature oddball paradigm directly subtract the ERPs in response to deviant and standard sounds and presume that their physical differences do not bias the MMN amplitude, latency, and/or topography. For sound intensity deviants, most studies acknowledge that larger exogenous potentials are elicited by louder sounds and use softer deviants compared to standards, therefore, however, underestimating the MMN amplitude. In traditional oddball paradigms, frequently reversed oddball blocks are included where the role of standard and deviant sounds is exchanged and deviants from the oddball blocks can be compared to physically identical standards from the reversed blocks. Also, the adaptation control conditions (see above) implicitly control for physical identity.
4. *No control for adaptation*. We did not find any peer‐reviewed publication employing the auditory multi‐feature oddball paradigm that appropriately controls for adaptation.^1^ Therefore, it appears to be presumed with the application of this multi‐feature oddball paradigm that the contribution of adaptation to the observed MMN is either negligible or at least constant across features and, more importantly, constant across comparisons between conditions and groups. This latter assumption is particularly important because adaptation and predictive processing may be modulated independently in specific conditions or groups. For example, predictive processing has been associated with N‐methyl‐D‐aspartate (NMDA) receptor function and has been reported to be impaired in patients suffering from schizophrenia. Therefore, differentiating the contributions of adaptation from predictive processing appears feasible. We will explore its implications in more detail in Section 4.

### 1.3 Aim and Hypotheses

Taken together, in the auditory multi‐feature paradigm, as it is commonly applied, it is presumed that *transition probability and context*, *integrated processing of sound features*, *physical differences*, and *adaptation* do not affect the MMN (amplitude, latency, and/or topography) or alternatively that their contribution to the observed MMN is negligible or *at least* constant across comparisons of features, conditions, or groups. We aimed to empirically test this assumption by controlling for *physical identity* and *adaptation*. To this end, we applied the multi‐feature cascadic control condition we previously developed for the visual MMN (Male et al. 2020) to the auditory modality. A multi‐feature oddball paradigm including frequency, location, intensity, and duration deviants and corresponding multi‐feature cascadic control condition blocks varying two features per block were measured within one session. We measured the classic and genuine MMN per feature by comparing ERPs in response to deviant sounds with the ERPs in response to standard and control sounds, respectively. We confirmed stimulus discriminability in an active condition. If all assumptions do hold, we expected similar classic (deviant minus standard) and genuine MMNs (deviant minus controls). Significant differences between the classic and genuine MMNs would indicate relevant contributions by *adaptation* and/or *physical difference*. Variable effects between sound *features* might indicate that they are not processed *independently*. Smaller or larger differences between the classic and genuine MMNs compared to the traditional oddball paradigm including the equiprobable or cascadic control conditions might indicate effects of *transitional probabilities and context*.

## 2 Method

### 2.1 Participants

We tested 32 self‐declared healthy adult participants. The data from two participants had to be excluded from the analysis: one due to technical problems and one because she/he fell asleep during the recording. All participants declared normal or corrected‐to‐normal vision and normal hearing. The mean age of the remaining participants was 24.2 years (SD = 4.9 years, range 18–39 years). Eighteen of the participants were female, 12 male; all participants were right‐handed. The local ethics committee approved the experiment. All participants provided their written informed consent and were free to withdraw from the experiment at any time. Participants received monetary compensation or course credits in return for participation.

### 2.2 Stimuli and Apparatus

Participants were comfortably seated in a dimly lit, electrically shielded, and sound‐attenuated chamber. In the passive part, they watched a self‐chosen silent subtitled movie on the display of a 17″ laptop in front of them. In the active part, a small white fixation cross was presented on a black background at the center of the display. Sounds were presented binaurally with headphones (Sennheiser HD25). In the active part, participants gave their responses by pressing a key on a four‐key response pad connected to a response registration device (RTBox; Li et al. 2010). A PC with Ubuntu Linux v16.04, Octave v4.0, and Psychophysics Toolbox 3.0.14 (Kleiner et al. 2007) presented sound stimuli and recorded responses.

The difference between deviant and standard sound stimulus parameters was roughly oriented on Pakarinen et al. (2007; corresponding to their levels “L3,” 23 Hz for frequency and 24 ms for duration—23.1 Hz and 25 ms here; between their levels “L2,” 5 dB SPL and “L3,” 7.5 dB SPL for intensity—6 dB SPL here; and between “L3,” 250 μs ITD; and “L4,” 400 μs ITD for location—312.5 μs ITD here; see Table 2) in order to be able to derive canonical MMN latencies for the ERP analysis mean amplitude time windows. In the multi‐feature oddball blocks, standards had a fundamental frequency of 523.3 Hz, an inter‐aural time difference (ITD) of −312.5 μs (approximately 45° front left), an intensity of 72 dB SPL, and a duration of 75 ms. Deviant sounds had either a fundamental frequency of 546.4 Hz, or an ITD of −625 μs (approximately 90° left), or an intensity of 78 dB SPL,^2^ or a duration of 50 ms (note that the figures are not corrected for the 50 ms delay in deviance onset for duration, but time 0 always reflects sound onset). The sound stimulus parameters used for the cascadic control blocks are displayed in Table 2. All sounds had a raised‐cosine window shaped rise time and fall time of 5 ms and included the second and third harmonics at an intensity of −3 and −6 dB, respectively.

**TABLE 2:** Stimulus parameters of the sounds used in the multi‐feature oddball paradigm (levels 1 and 2; shaded in gray) and the multi‐feature cascadic control paradigm (Levels 1–5).

	Feature			
Feature level	Frequency	Location (ITD)	Intensity	Duration
Level 1 (multi‐feature deviant)	546.4 Hz	−625 μs	78 dB SPL	50 ms
Level 2 (multi‐feature standard)	523.3 Hz	−312.5 μs	72 dB SPL	75 ms
Level 3	501.1 Hz	0 μs	66 dB SPL	112 ms
Level 4	479.8 Hz	312.5 μs	60 dB SPL	168 ms
Level 5	459.5 Hz	625 μs	54 dB SPL	253 ms

### 2.3 Procedure

The experiment started with a passive part of 60‐min duration (excluding breaks) including 18 blocks during which participants watched a silent subtitled movie. It was followed by an active part of approximately 15‐min duration (excluding breaks) including three blocks during which participants were asked to discriminate as correctly as possible whether two sequentially presented sounds were the same or different. During all blocks, participants were asked to move as little as possible and blink normally. Participants were free to take short breaks between blocks.

The 18 blocks of the passive part consisted of six multi‐feature oddball blocks and 12 cascadic control blocks presented in randomized order. In each block, there were 400 stimuli presented with a stimulus‐onset asynchrony (SOA) of 0.5 s, resulting in a block length of 200 s and a total duration of the passive part of 60 min. In the multi‐feature blocks, standard and deviant stimuli appeared predictably on alternate trials (Table 1). Each deviant feature appeared once per set of four standard/deviant pairs of trials in pseudo‐randomized order with the constraint that the same deviant feature never appeared in two subsequent pairs of trials. The probability that each deviant feature occurred in a deviant trial was 25%. Hence, for each set of eight stimuli, for each sound feature, the deviant feature was presented in one out of eight trials (12.5%) and the standard feature in seven out of eight trials (87.5%; similar to the traditional oddball paradigm; see above). In each of the multi‐feature blocks, there were 200 standards and 200 deviants (50 per sound feature), resulting in 300 deviants per sound feature in the six blocks in total.

In the cascadic control blocks (illustrated in Table 3), we interspersed control stimuli within a block, which were physically identical to the deviant stimuli in the multi‐feature oddball blocks. The relevant stimulus feature was varied (see Table 3) in a regular descending and ascending sequence (e.g., 5–4–3–2–1–2–3–4). Ideally, we would have included all four sound features concurrently in the cascadic control blocks. However, this was not possible without sacrificing the physical identity constraint of the deviant and control stimuli. That is, for each of the four relevant cascadic control sounds, the controlled feature should be identical to the corresponding multi‐feature deviant, and the remaining three features should be identical to the multi‐feature standard. However, with four interleaved feature cascades for two of the features, the level would differ from that of the multi‐feature standard. As we considered the physical identity constraint to be more important in the context of our research question, we combined two features per cascadic control block. All six possible combinations of the four manipulated sound features (frequency, location, intensity, and duration) were used in two of the blocks in order to cover all possible effects of feature integration. The two‐feature cascades had an offset of one trial: Each feature led once in one of two blocks, resulting in 12 cascadic control blocks. This design allowed the corresponding standard feature to always precede each control stimulus. In each of the cascadic control blocks, there were 50 control stimuli per feature, resulting in 300 control stimuli per sound feature in all blocks in total. Example sound files for the multi‐feature condition (mf.wav) and the cascading control condition for the combinations of frequency and location (mf_casc_fl.wav) and frequency and duration (mf_casc_fd.wav) can be downloaded from the supplementary materials provided in the OSF repository (https://osf.io/adh3j/).

**TABLE 3:** Illustration of the auditory multi‐feature cascadic control condition. Sound features that are identical to standard sound features in the multi‐feature paradigm are marked in italics. Sound features that are identical to deviant sound features in the multi‐feature paradigm are marked in bold. Two sound features are varied in a regular descending and ascending sequence (here, frequency, marked in blue and location, marked in red; all possible feature combinations were used in different blocks). Two sound features are kept constant per block (here, intensity and duration). The two control sounds for location (Trial 4; shaded in gray) and frequency (Trial 5) are physically identical to the corresponding deviants in the multi‐feature oddball paradigm (see Table 1, Trials 2 and 4). The trial directly preceding the control sound always has the same feature level (L2/F2) as the standards in the multi‐feature oddball paradigm to better equalize the adaptational state.

Feature level	Trial 1	Trial 2	Trial 3	Trial 4	Trial 5	Trial 6	Trial 7	Trial 8
Level 5	F5							L5
Level 4	L4	F4					L4	F4
Level 3		L3	F3			L3	F3	
*Level 2* (*multi‐feature standard*)	*I2 D2*	*I2 D2*	*L2 I2 D2*	*F2 I2 D2*	*L2 I2 D2*	*F2 I2 D2*	*I2 D2*	*I2 D2*
**Level 1 (multi‐feature deviant)**				**L1**	**F1**			

The active task was presented to examine whether participants were able to discriminate standard from deviant feature levels for all four sound features (Table 2). The active part started with a written instruction and consisted of three blocks lasting approximately 4–6 min each. We asked participants to fixate a fixation cross that was always present. In a two‐interval, two‐alternative, forced choice task, we asked participants to listen to sounds and to judge whether two successively presented sounds were the same or different. The sound pairs were presented with an SOA of 0.5 s. The next trial started 0.5 s after the response. There were 128 pairs per block; 64 pairs were the same (both standard sounds from the multi‐feature oddball paradigm), and 64 pairs were different, one standard and one deviant sound from the multi‐feature oddball paradigm (16 per deviant feature). The order was balanced between the first and second interval. Participants responded by pressing the “same” button with the index finger of one hand and the “different” button with the index finger of the other hand (counterbalanced across participants). The button assignment was displayed on the screen below the fixation cross. There was no reaction time limit.

### 2.4 EEG Recording

We recorded the electroencephalogram (EEG) from 28 Ag/AgCl active electrodes attached to an electrode cap (actiCAP). We placed electrodes at Fp1, Fp2, F7, F3, Fz, F4, F8, FC5, FC1, FC2, FC6, T7, C3, Cz, C4, T8, CP5, CP1, CP2, CP6, P7, P3, Pz, P4, P8, and Oz according to the extended international 10–20 system and at the left and right mastoids. We recorded EEG at a 500 Hz sampling rate with an actiCHamp EEG amplifier (Brain Products, Gilching, Germany). We recorded the electrooculogram (EOG) from electrodes placed at the outer canthi of both eyes and an electrode placed below the left eye. Impedances were kept below 20 kΩ. We placed the ground electrode at the Fpz electrode location and the reference electrode on the nose tip.

### 2.5 EEG Data Analysis

EEG data analysis was performed with MATLAB software and the EEGLAB toolbox (Delorme and Makeig 2004). Data were filtered offline with a 0.1‐Hz high‐pass filter (−6 dB cutoff, zero‐phase Hamming windowed sinc FIR filter, order = 8250, transition width = 0.2 Hz; Widmann and Schröger 2012; Widmann et al. 2015) and a 48‐Hz low‐pass filter (−6 dB cutoff, zero‐phase Hamming windowed sinc FIR filter, order = 414, transition width = 4 Hz). The data were segmented into epochs of 0.5‐s duration including a 0.1‐s pre‐stimulus baseline. The robust standard deviation was computed per channel (0.7413 times the interquartile range). Noisy channels with a robust z‐score (the median centered channel robust standard deviation divided by 0.7413 times the interquartile range of the channel robust standard deviations) of the robust standard deviation larger than 3 were removed from the data (a single channel in three and two channels in one of the participants; Bigdely‐Shamlo et al. 2015). We also excluded epochs including amplitude differences exceeding 750 μV at any channel in order to remove large non‐stereotypical artifacts but to keep stereotypical artifacts as blinks and eye movements to be later removed using independent component analysis (ICA). Data were corrected for eye movement artifacts using ICA. To improve the decomposition, ICA was computed on the raw data (excluding bad trials and channels) filtered by a 1‐Hz high‐pass filter (−6 dB cutoff, zero‐phase Hamming windowed sinc FIR filter, order = 1650, transition band width = 1 Hz) and a 48‐Hz low‐pass filter (see above) and segmented into epochs of 0.5 s but not baseline corrected (Groppe et al. 2009). The obtained demixing matrix was then applied to the 0.1–48 Hz filtered data. It has been validated that high‐pass filters improve ICA decompositions (Klug and Gramann 2021) and the demixing matrix can be applied to a linearly transformed data set (Winkler et al. 2015). ICA components were classified by the ICLabel EEGLAB plug‐in for automatic independent component (IC) classification, manually selected, and pruned (Pion‐Tonachini et al. 2019). Component rejection was restricted to heart beat and eye movement–related ICA components, that is, blinks, horizontal and vertical pre‐saccadic spike potential, horizontal, and vertical movements of the corneo‐retinal dipole and blink/eyelid‐induced artifacts (Plöchl et al. 2012). On average, 5.3 components per participant were eliminated (range of 5–6 components). The topographies of all rejected components are shown in Figures S1 (participants 1–10), S2 (participants 11–20), and S3 (participants 21–30) in the supplementary materials provided in the OSF repository (https://osf.io/adh3j/). Subsequently, trials with amplitude differences exceeding 150 μV at any channel and the first eight trials per block were excluded from the analysis. Individual average ERPs were computed per participant, condition, and stimulus type. Grand‐average waveforms were computed on the basis of the individual averages (the number of included trials per condition is described in Table 4).

**TABLE 4:** Mean, median, standard deviation, and range of numbers of trials included per stimulus type.

Stimulus type	M	Mdn	SD	Min	Max
Multi‐feature oddball paradigm					
Standard	1162.4	1169.5	17.5	1113	1176
Frequency deviant	291.0	293	3.5	281	294
Location deviant	290.8	292.5	3.9	280	294
Intensity deviant	290.8	293	4.3	279	294
Duration deviant	290.6	293	4.8	275	294
Cascadic control condition					
Frequency control	290.4	292	5.1	270	294
Location control	290.8	293	6.2	263	294
Intensity control	291.2	293	4.5	274	294
Duration control	290.9	292	4.7	270	294

### 2.6 ERP Quantification and Statistical Analysis

**FIGURE 1:**
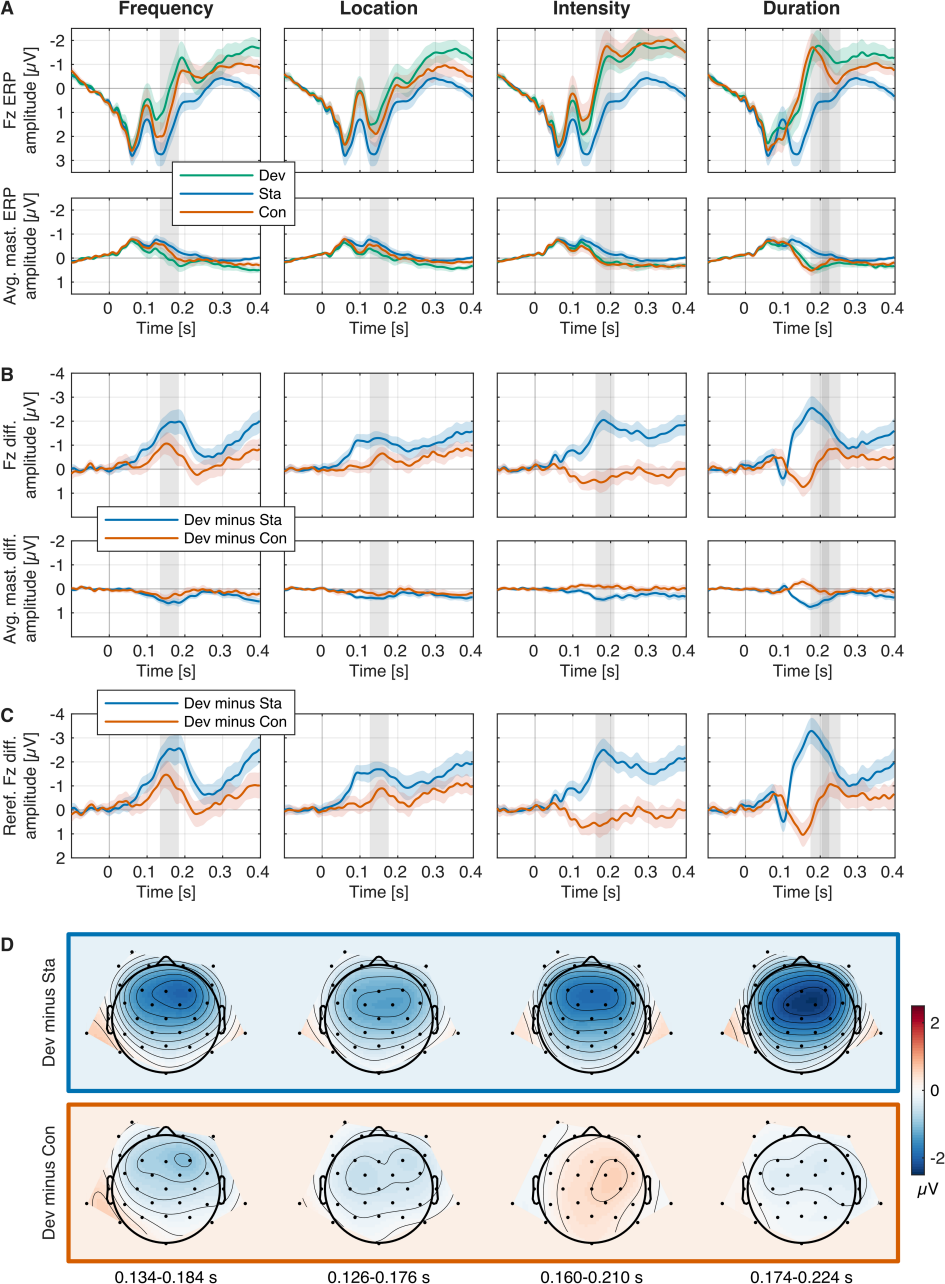
Panel A: ERPs for standard (Sta; blue), deviant (Dev; green), and control sounds (Con; red) per deviant feature at Fz and averaged mastoids electrode locations. Panels B and C: Corresponding deviant minus standard (blue; classic MMN) and deviant minus control difference waves (red; genuine MMN). Panel C: Corresponding difference waves at Fz electrode location re‐referenced to averaged mastoids. Shaded areas indicate 95% confidence intervals. Analysis time windows are marked with gray bars and were canonically derived (Pakarinen et al. 2007). For duration additionally an adjusted time window (0.204–0.254 s) centered on the peak of the average mastoid re‐referenced deviant minus control difference wave at Fz was examined. Panel D shows deviant minus standard and deviant minus control topographies within the analysis time windows.

**FIGURE 2:**
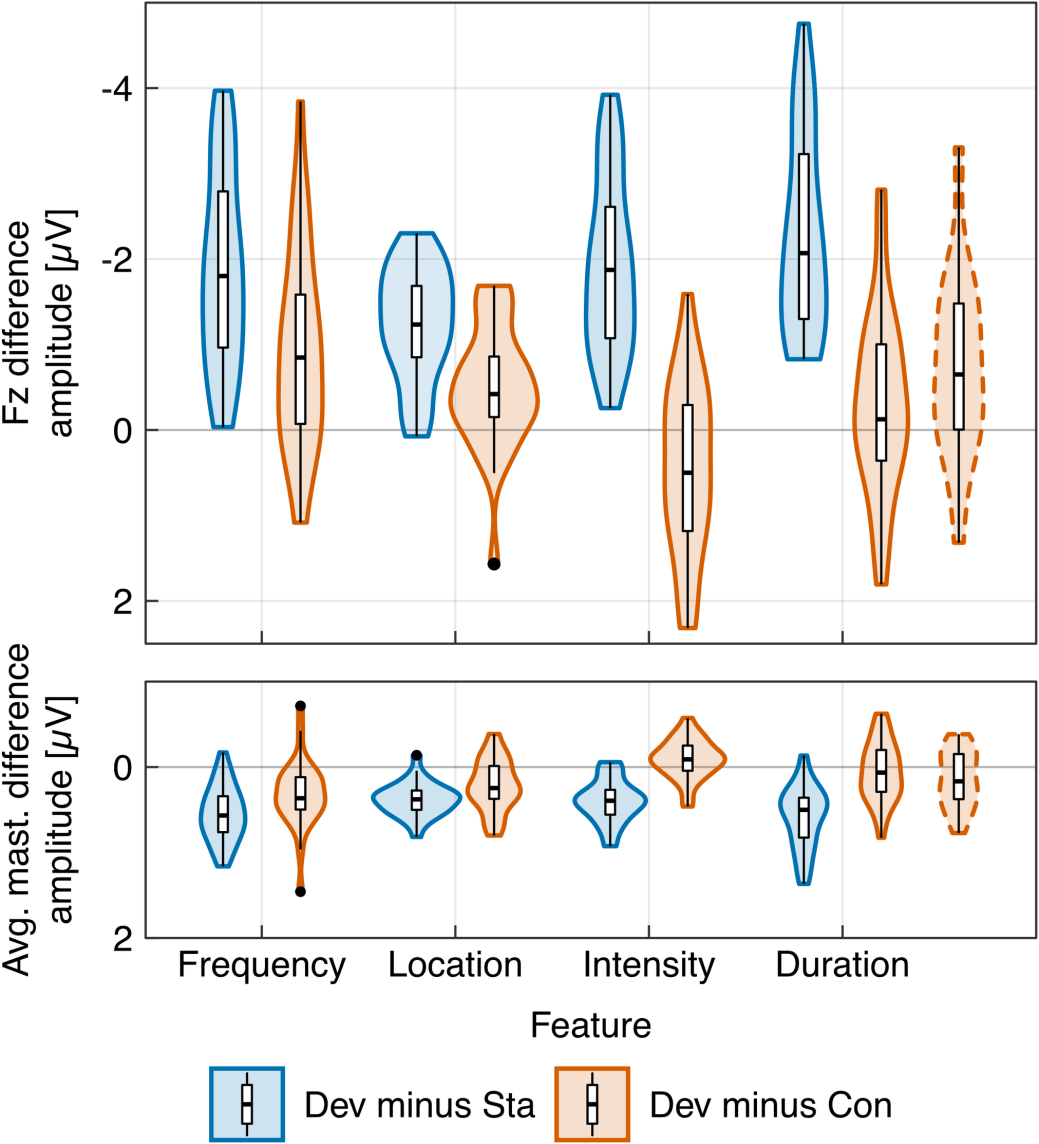
Violin and box plots illustrating the distributions of the deviant (Dev) minus standard (Sta) classic (blue) and deviant minus control (Con) genuine MMN (red) mean amplitudes per feature at Fz electrode location and averaged mastoids (adjusted mean amplitude time window for duration deviants with dashed outline).

Mean amplitude time windows for ERP analysis were canonically derived from Pakarinen et al. (2007): Time windows of 50 ms duration were centered on the MMN peak latency as displayed in Pakarinen et al. (2007; figure 2; Fz electrode location re‐referenced to average mastoids for the corresponding feature and magnitude of deviation) (“L3” for frequency and duration; linear interpolation was used for location and intensity). The resulting time windows were 0.134–0.184 s for frequency, 0.126–0.176 s for location, 0.160–0.210 s for intensity, and 0.174–0.224 s for duration (relative to stimulus onset, 0.124–0.174 relative to the onset of the deviation for duration deviants). We would like to note that except for duration (see below for discussion) grand‐average peak‐centered time windows would not have substantially differed from the canonically derived time windows (the average mastoid re‐referenced difference waves are displayed in Figure 1, Panel C, for evaluation). ERP time window mean amplitudes were computed at Fz and averaged mastoid electrode locations per participant, condition, and stimulus type.

We compared mean amplitudes at Fz and averaged mastoids electrode locations between deviant and standard as well as between deviant and control stimuli per feature with directed (one‐tailed) Bayesian and frequentist *t*‐tests (Table 5). Bayesian *t*‐tests were computed in JASP (Version 0.19.3 JASP Team 2025; Van Den Bergh et al. 2023) and the default scaling factor *r* = 0.707 (corresponding to the default “medium” effect size prior in the R BayesFactor package; Morey and Rouder 2012).

We computed identical 2 × 4 Bayesian and frequentist repeated measures ANOVA designs on the difference amplitudes with the factors *condition* (deviant minus standard vs. deviant minus control) and *feature* (frequency vs. location vs. intensity vs. duration). Statistically significant interaction effects were analyzed with two‐tailed follow‐up Bayesian and frequentist *t*‐tests. Bayesian ANOVAs were computed in JASP and the default scaling factor *r* = 0.5 for fixed effects (equivalent to the effect size scaling parameter used for the *t*‐tests and corresponding to the default “medium” effect size prior for fixed effects in the R BayesFactor package) and *r* = 1 for the participant random effect (corresponding to the default “nuisance” prior for random effects in the R BayesFactor package). We compared all models (constrained by the principle of marginality) with the null model (BF_10_) and additionally evaluated main effects and interactions by comparing the averaged models containing a main effect or interaction to the averaged matched models stripped of the effect (BF_Incl_). Data were interpreted as moderate evidence in favor of the alternative (or null) hypothesis if BF_10_ was larger than 3 (or lower than 0.33), strong evidence if BF_10_ was larger than 10 (lower than 0.1), or very strong evidence if BF_10_ was larger than 30 (lower than 0.033, Lee and Wagenmakers 2014). BF_10_ between 0.33 and 3 is considered as weak evidence (termed “anecdotal evidence” by Lee and Wagenmakers 2014).

An alpha level of 0.05 was defined for all frequentist tests. Results were reported including the generalized *η* ^2^_G_ (ANOVAs) or Cohen's *d*_*z*_ (*t*‐tests) effect size measures. We employed the Greenhouse–Geisser correction for degrees of freedom where appropriate.

The d‐prime sensitivity index (d′) was computed per participant and sound feature from the hit rate (proportion of correct “different” responses in trials with different feature levels in the two intervals) and false alarm rate (proportion of incorrect “different” responses in trials with identical feature levels in the two intervals) in the discriminability blocks in the active part. We applied log‐linear correction of individual hit and false alarm rates (Stanislaw and Todorov 1999).

We compared the effect sizes of the amplitude difference between the classic and genuine MMN in the present study with the corresponding effect sizes in traditional oddball MMN studies (where available) using the Equality‐of‐Effect‐Size Bayes factor (Verhagen and Wagenmakers 2014) and the meta‐analytic *Q* test (Borenstein et al. 2021).

## 3 Results

### 3.1 Behavioral Data

Sensitivity was very good for all deviant features (frequency: d′ = 3.75, SD = 0.62; location: d′ = 2.51, SD = 0.96; intensity: d′ = 3.38, SD = 0.71; duration: d′ = 2.89, SD = 0.79), suggesting that participants were able to detect deviants with high accuracy.

### 3.2 ERP Data

ERPs for standard, deviant, and control sounds and deviant minus standard and deviant minus control difference waves and topographies are displayed per deviant feature in Figure 1. The classic and genuine MMN distributions are illustrated per deviant feature in Figure 2. Deviant minus standard (classic MMN) and deviant minus control (genuine MMN) mean amplitudes and effect sizes in the respective time windows were reported in Table 5 together with deviant vs. standard and deviant vs. control Bayesian and frequentist directed *t*‐tests per feature.

The data provide very strong evidence for a classic MMN (deviant minus standard) elicited by all feature deviants inverting polarity over mastoidal leads (all *BF*_10_ > 100; see Table 5 for details). The classic MMN peak latency fell within the canonically derived analysis time window for all deviant features. The data also provide very strong evidence for a genuine MMN (deviant minus control) elicited by frequency and location deviants, however, with smaller amplitudes compared to the classic MMN amplitudes by about a factor of 2 (frequency) to 3 (location) and also inverting polarity over mastoidal leads (all *BF*_10_ > 30). The data provide strong evidence against an observation of a genuine MMN by intensity deviants (but rather a small positivity at Fz and a very small negative deflection at mastoidal electrode locations; all *BF*_10_ < 0.1). The data do not provide conclusive evidence for or against an observation of a genuine MMN for duration deviants within the canonical analysis time window (*BF*_10_ ~ 1). A later negative peak (242 ms latency at Fz) was observed in the deviant minus control difference wave for duration deviants accompanied by a very small polarity inversion over mastoidal leads (peak latency 228 ms). In an adjusted 0.204–0.254 s mean amplitude time window (centered on the peak of the average mastoid re‐referenced deviant minus control difference wave at Fz), the data provided strong evidence for the observation of a genuine MMN at Fz (BF_10_ > 30) and moderate evidence at mastoidal leads (BF_10_ > 3).

#### 3.2.1 ANOVAs Fz

We observed more negative amplitudes of the classic MMN (deviant minus standard) compared to the genuine MMN (deviant minus control) for all features at the Fz electrode location. The amplitude difference between the classic and genuine MMN significantly varied between features. The Bayesian ANOVA on the MMN amplitudes preferred the full model (*BF*_10_ = 6.29 × 10^24^) including both the feature (*BF*_Incl_ = 9.02; *F*(3,87) = 4.61, *p* = 0.009, *ε* = 0.812, *η* ^2^_G_ = 0.072) and condition (*BF*_Incl_ = 3.4 × 10^10^; *F*(1,29) = 170.12, *p* < 0.001, *η* ^2^_G_ = 0.368) main effects and their interaction (*BF*_Incl_ = 2.26 × 10^13^; *F*(3,87) = 39.38, *p* < 0.001, *ε* = 0.877, *η* ^2^_G_ = 0.102). The data provide very strong evidence for more negative amplitudes of the classic MMN vs. genuine MMN for all features (all BF_10_ > 100, all *p* < 0.001; exact details of the corresponding follow‐up tests are reported in Table 5, highlighted by gray shading). Frequentist and Bayesian *t*‐tests that compare the difference of classic and genuine MMN between features are available in JASP and html formats in the OSF repository (https://osf.io/adh3j/).

**TABLE 5:** Deviant vs. standard (classic MMN) and deviant vs. control (genuine MMN) time window (TW) mean amplitudes, effect sizes (Cohen's *d*_*z*_; numbers in square brackets indicate 95% confidence intervals), and Bayesian and frequentist *directed t*‐tests per feature and region of interest (ROI; Fz and averaged mastoids) and the corresponding classic vs. genuine MMN Bayesian and frequentist *two‐sided t*‐tests (shaded in gray).

ROI	Feature	TW [s]	Mean [μV]	BF_−0_	*t*(29)	*p*	*d*_*z*_
Fz	Deviant minus standard (classic MMN)						
	Frequency	0.134–0.184	−1.87 [−2.30, −1.44]	2.47 × 10^7^	−8.88	< 0.001	−1.62 [−2.16, −1.07]
	Location	0.126–0.176	−1.25 [−1.50, −1.00]	5.82 × 10^8^	−10.32	< 0.001	−1.88 [−2.48, −1.28]
	Intensity	0.160–0.210	−1.91 [−2.29, −1.53]	5.90 × 10^8^	−10.32	< 0.001	−1.89 [−2.48, −1.28]
	Duration	0.174–0.224	−2.29 [−2.72, −1.86]	1.68 × 10^9^	−10.82	< 0.001	−1.98 [−2.59, −1.35]
	Deviant minus control (genuine MMN)						
	Frequency	0.134–0.184	−0.91 [−1.35, −0.47]	245.03	−4.22	< 0.001	−0.77 [−1.17, −0.36]
	Location	0.126–0.176	−0.49 [−0.76, −0.22]	84.21	−3.76	< 0.001	−0.69 [−1.08, −0.28]
	Intensity	0.160–0.210	0.44 [0.07, 0.81]	0.06	2.42	0.989	0.44 [0.06, 0.81]
	Duration	0.174–0.224	−0.30 [−0.70, 0.09]	1.09	−1.57	0.064	−0.29 [−0.65, 0.08]
	Duration adj. TW	0.204–0.254	−0.75 [−1.15, −0.35]	92.69	−3.80	< 0.001	−0.69 [−1.09, −0.29]
	Classic vs. genuine MMM			BF_10_			
	Frequency	0.134–0.184	−0.96 [−1.28, −0.65]	2.58 × 10^4^	−6.32	< 0.001	−1.15 [−1.61, −0.68]
	Location	0.126–0.176	−0.76 [−0.99, −0.52]	5.37 × 10^4^	−6.61	< 0.001	−1.21 [−1.67, −0.73]
	Intensity	0.160–0.210	−2.35 [−2.74, −1.96]	1.66 × 10^10^	−12.34	< 0.001	−2.25 [−2.93, −1.57]
	Duration	0.174–0.224	−1.98 [−2.32, −1.65]	1.04 × 10^10^	−12.09	< 0.001	−2.21 [−2.87, −1.53]
	Duration adj. TW	0.204–0.254	−1.54 [−1.84, −1.24]	3.11 × 10^8^	−10.35	< 0.001	−1.89 [−2.49, −1.28]
Avg. mast.	Deviant minus standard (classic MMN)			BF_+0_			
	Frequency	0.134–0.184	0.55 [0.43, 0.67]	4.73 × 10^7^	9.17	< 0.001	1.67 [1.11, 2.23]
	Location	0.126–0.176	0.38 [0.31, 0.45]	1.22 × 10^9^	10.67	< 0.001	1.95 [1.33, 2.56]
	Intensity	0.160–0.210	0.40 [0.31, 0.49]	1.87 × 10^7^	8.76	< 0.001	1.60 [1.05, 2.14]
	Duration	0.174–0.224	0.59 [0.45, 0.73]	2.08 × 10^7^	8.80	< 0.001	1.61 [1.06, 2.15]
	Deviant minus control (genuine MMN)						
	Frequency	0.134–0.184	0.32 [0.17, 0.47]	386.03	4.39	< 0.001	0.80 [0.38, 1.21]
	Location	0.126–0.176	0.21 [0.10, 0.33]	104.82	3.85	< 0.001	0.70 [0.30, 1.10]
	Intensity	0.160–0.210	−0.09 [−0.18, −0.01]	0.07	−2.21	0.982	−0.40 [−0.77, −0.03]
	Duration	0.174–0.224	0.06 [−0.07, 0.19]	0.46	0.91	0.186	0.17 [−0.20, 0.52]
	Duration adj. TW	0.204–0.254	0.15 [0.02, 0.15]	4.79	2.44	0.010	0.45 [0.07, 0.82]
	Classic vs. genuine MMM			BF_10_			
	Frequency	0.134–0.184	0.23 [0.11, 0.35]	48.19	3.82	< 0.001	0.70 [0.29, 1.09]
	Location	0.126–0.176	0.17 [0.08, 0.25]	91.34	4.08	< 0.001	0.75 [0.34, 1.15]
	Intensity	0.160–0.210	0.49 [0.40, 0.58]	2.54 × 10^9^	11.37	< 0.001	2.08 [1.43, 2.71]
	Duration	0.174–0.224	0.53 [0.41, 0.65]	1.85 × 10^7^	9.06	< 0.001	1.65 [1.09, 2.20]
	Duration adj. TW	0.204–0.254	0.44 [0.34, 0.54]	1.06 × 10^7^	8.81	< 0.001	1.61 [1.06, 2.15]

#### 3.2.2 ANOVAs Averaged Mastoids

We observed more positive amplitudes for the classic MMN (deviant minus standard) compared to the genuine MMN (deviant minus control) for all features at averaged mastoid electrode locations. The amplitude difference between the classic and genuine MMN significantly varied between features. The Bayesian ANOVA on the MMN amplitudes preferred the full model (*BF*_10_ = 2.09 × 10^16^) including both the feature (*BF*_Incl_ = 24.38; *F*(3,87) = 5.53, *p* = 0.004, *ε* = 0.810, *η* ^2^_G_ = 0.096) and condition (*BF*_Incl_ = 4.47 × 10^7^; *F*(1,29) = 91.69, *p* < 0.001, *η* ^2^_G_ = 0.254) main effects and their interaction (*BF*_Incl_ = 1.96 × 10^7^; *F*(3,87) = 19.94, *p* < 0.001, *ε* = 0.957, *η* ^2^_G_ = 0.064). The data provide very strong evidence for more positive amplitudes of the classic MMN vs. genuine MMN for all features (all BF_10_ > 30, all *p* < 0.001; exact details of the corresponding follow‐up tests are reported in Table 5, highlighted by gray shading). Frequentist and Bayesian *t*‐tests that compare the difference of classic and genuine MMN between features are available in JASP and html formats in the OSF repository (https://osf.io/adh3j/).

#### 3.2.3 The Classic vs. Genuine MMN in the Multi‐Feature and the Traditional Oddball Paradigm

The data did not provide conclusive evidence for or against a difference in the effect size of the classic minus genuine *frequency* MMN amplitude between the present study (Fz; *d*_*z*_ = −1.15) and the traditional oddball *frequency* MMN studies by Maess et al. (2007) (*d*_*z*_ = −0.66; *BF*_01_ = 0.588; *Q*(1) = 1.76, *p* = 0.185) and Wiens et al. (2019) (*d*_*z*_ = −0.64; *BF*_01_ = 0.845; *Q*(1) = 2.58, *p* = 0.108). The data provided strong evidence for a larger difference in the effect size of the classic minus genuine *frequency* MMN amplitude in the present study (Fz; *d*_*z*_ = −1.15) than the traditional oddball *frequency* MMN study by Ruhnau et al. (2012) (cascading: *d*_*z*_ = −0.04; *BF*_01_ = 52.9; *Q*(1) = 10.51, *p* = 0.001; equiprobable: *d*_*z*_ = −0.21; *BF*_01_ = 10.5; *Q*(1) = 7.36, *p* = 0.007). The data did not provide conclusive evidence for or against a difference in the effect size of the classic minus genuine *location* MMN amplitude between the present study (Fz; *d*_*z*_ = −1.21) and the traditional oddball *location* MMN study by Schröger and Wolff (1996) (*d*_*z*_ = −0.66; *BF*_01_ = 0.667; *Q*(1) = 1.9, *p* = 0.168). The data provided moderate and strong evidence for a larger difference in the effect size of the classic minus genuine *duration* MMN amplitude in the present study (Fz, adjusted time window; *d*_*z*_ = −1.89) than the traditional oddball *duration* MMN study by Hsu et al. (2010) (*d*_*z*_ = −0.89; *BF*_01_ = 3.44; *Q*(1) = 4.30, *p* < 0.038) and Jacobsen and Schröger (2003) (*d*_*z*_ > −0.32; *BF*_01_ = 267; *Q*(1) = 12.45, *p* < 0.001), respectively.

## 4 Discussion

We performed an auditory multi‐feature oddball paradigm including standards and frequency, location, intensity, and duration deviants. We additionally designed and performed a multi‐feature cascadic control paradigm including control stimuli physically identical to and equally adapted as the corresponding deviant stimuli. We observed a reliable classic MMN—a negative difference in the ERPs in response to deviant minus standard sounds over frontal leads inverting polarity over mastoidal leads—in response to all deviant features in the multi‐feature oddball paradigm. We observed a genuine MMN—comparing the multi‐feature oddball deviant sounds to the multi‐feature cascadic control sounds—with considerably smaller amplitude by a factor of two to three compared to the classic MMN in response to frequency and location deviants. We did not find a genuine MMN in response to intensity and duration deviants (but only a considerably delayed and smaller negative deflection in the case of duration deviants only minimally inverting polarity). Deviant and standard sounds could be discriminated by participants with high precision.

We developed a new control condition for the multi‐feature oddball paradigm in the auditory modality (see Male et al. 2020 for the visual modality) controlling for physical difference and adaptation. The cascadic control condition successfully enabled the separation of the genuine MMN from other deviance‐sensitive processes such as adaptation. We consider this separation as highly relevant for the investigation of genuine deviant detection and prediction mechanisms. In the following, we discuss the different relative contributions of (stimulus‐specific) adaptation and memory or prediction‐based higher order deviance detection between the multi‐feature and the traditional oddball paradigms and between sound features. We will conclude with a discussion of theoretical implications and the relevance of our findings for the investigation of mechanisms underlying the MMN in clinical or other sensitive populations using the multi‐feature paradigm.

### 4.1 The Genuine MMN in the Traditional and the Multi‐Feature Oddball Paradigms

In response to frequency, location, and duration (in an adjusted time window only) deviants, the observed genuine MMN were smaller by a factor of two to three than the classic MMN (in terms of amplitudes as well as in terms of effect sizes; cf., Table 5). In response to intensity and duration (in the canonical time window) deviants, the genuine MMN disappeared when controlling for adaptation and physical differences. We aimed to compare the *difference* between the classic and the genuine MMN amplitudes between the multi‐feature oddball paradigm as reported here and those observed in the traditional oddball paradigm including an equiprobable or cascadic control condition. To this end, we selected a set of studies explicitly examining one or more of the adaptation control conditions and including the same features in a range comparable to the present study (note that the selection of included studies was not necessarily exhaustive). A summary of the relevant statistical parameters and corresponding effect sizes is reported in Table 6.

**TABLE 6:** Effect size estimates (Cohen's *d*_*z*_) of the classic (Dev minus Sta) and genuine MMN (Dev minus Con) observed in the traditional oddball paradigm applying the equiprobable or cascadic adaptation control paradigm for deviations of frequency, location, intensity, and duration. Effect sizes for the amplitude difference between the classic and genuine MMN in gray shading (where reported). Time windows are reported relative to deviation onset.

Contrast	ROI	Time window	N	*F*	*t*	*d*_*z*_
Jacobsen and Schröger (2001), frequency						
Dev minus Sta	Fz	170–190 ms	8	18.8	−4.34	−1.53
Dev minus Con	Fz	170–190 ms	8	5.9	−2.43	−0.86
Dev minus Sta	Avg. mast.	170–190 ms	8	25.5	5.05	1.79
Dev minus Con	Avg. mast.	170–190 ms	8	10.0	3.16	1.12
Maess et al. (2007), frequency, MEG						
Dev minus Sta vs. Dev minus Con	Loadings		15	6.47	−2.54	−0.66
Ruhnau et al. (2012), frequency						
Dev minus Sta	Fronto‐central cluster	140–190 ms	16	29.86	−5.46	−1.37
Dev minus Con_casc_	Fronto‐central cluster	140–190 ms	16	36.94	−6.08	−1.52
Dev minus Con_equi_	Fronto‐central cluster	140–190 ms	16	20.25	−4.50	−1.13
Dev minus Sta	Avg. mast.	140–190 ms	16	25.90	5.09	1.27
Dev minus Con_casc_	Avg. mast.	140–190 ms	16	33.63	5.80	1.45
Dev minus Con_equi_	Avg. mast.	140–190 ms	16	11.64	3.41	0.85
Dev minus Sta vs. Dev minus Con_casc_	Fronto‐central cluster	140–190 ms	16		−0.15	−0.04
Dev minus Sta vs. Dev minus Con_equi_	Fronto‐central cluster	140–190 ms	16		−0.85	−0.21
Dev minus Sta vs. Dev minus Con_casc_	Avg. mast.	140–190 ms	16		1.25	0.31
Dev minus Sta vs. Dev minus Con_equi_	Avg. mast.	140–190 ms	16		1.69	0.42
Wiens et al. (2019), frequency						
Dev minus Sta	Fronto‐central cluster	115–165 ms	26		−8.66	−1.70
Dev minus Con_casc_	Fronto‐central cluster	115–165 ms	26		−4.17	−0.82
Dev minus Con_norep_	Fronto‐central cluster	115–165 ms	26		−3.94	−0.77
Dev minus Sta vs. Dev minus Con_norep_	Fronto‐central cluster	115–165 ms	26		−3.24	−0.64
Dev minus Con_norep_ vs. Dev minus Con_casc_	Fronto‐central cluster	115–165 ms	26		−0.06	−0.01
Schröger and Wolff (1996), location						
Dev minus Sta	Fz reref. avg. mast.	165–215 ms	12		−7.31	−2.11
Dev minus Con	Fz reref. avg. mast.	190–240 ms	12		−2.89	−0.83
Dev minus Sta vs. Dev minus Con	Fz, Cz, Pz, L1, R1	165–215 ms vs. 190–240 ms (!)	12	5.20	−2.28	−0.66
Jacobsen et al. (2003), intensity						
Dev minus Sta softer	Fz	190–210 ms	10	10.19	−3.19	−1.01
Dev minus Sta_rev_ softer	Fz	188–208 ms	10	9.03	−3.00	−0.95
Dev minus Con softer	Fz	194–214 ms	10	12.82	−3.58	−1.13
Dev minus Sta louder	Fz	156–176 ms	10	9.86	−3.14	−0.99
Dev minus Sta_rev_ louder	Fz	154–174 ms	10	23.97	−4.90	−1.55
Dev minus Con louder	Fz	179–199 ms	10	5.20	−2.28	−0.72
Jacobsen and Schröger (2003), duration						
Dev minus Sta	Fz, Cz, Pz	130–150 ms	10	13.79	−3.71	−1.17
Dev minus Sta_rev_	Fz	140–160 ms	10	46.09	−6.79	−2.15
Dev minus Con	Fz	140–160 ms	10	15.00	−3.87	−1.22
Dev minus Sta	Avg. mast.	130–150 ms	10	8.52	2.92	0.92
Dev minus Sta_rev_	Avg. mast.	140–160 ms	10	25.42	5.04	1.59
Dev minus Con	Avg. mast.	140–160 ms	10	20.33	4.51	1.43
Dev minus Sta_rev_ vs. Dev minus Con	Fz	140–160 ms	10	< 1	> − 1	> −0.32
Dev minus Sta_rev_ vs. Dev minus Con	Avg. mast.	140–160 ms	10	< 1	< 1	< 0.32
Hsu et al. (2010), duration, MEG						
Dev minus Sta	LH, RH	130–200 ms	12		10.15	2.93
Dev minus Con	LH, RH	130–200 ms	11		9.72	2.93
Dev minus Sta vs. Dev minus Con	LH, RH	130–200 ms	10	8.0	−2.83	−0.89

Firstly, in contrast to the multi‐feature oddball paradigm (with cascadic control condition), in the traditional oddball paradigm (with equiprobable control condition), a genuine MMN was observed also for intensity and duration. Secondly, some studies statistically compared the classic and genuine MMN amplitudes directly. We computed effect size estimates for these direct comparisons that are highlighted by gray shading in both Tables 5 and 6, respectively. Note that these direct comparisons only include the within‐subject variance and can therefore be better compared between conditions and studies, in contrast to the classic and genuine MMN effect size estimates also including between‐subject variance. The reported effect sizes for the *difference* between the classic and genuine MMN obtained in these traditional oddball studies were considerably and consistently smaller (in magnitude) than the ones we observed in the present study in the multi‐feature oddball paradigm for all sound features. For example, for frequency deviants, the effect size estimate of the difference between the classic and genuine MMN amplitudes ranges from *d*_*z*_ = −0.66 (Maess et al. 2007) to *d*_*z*_ = −0.04 (Ruhnau et al. 2012) in the traditional oddball paradigm vs. *d*_*z*_ = −1.15 in the multi‐feature oddball paradigm (present study). When compared with the frequency MMN study by Ruhnau et al. (2012) and the duration MMN studies by Jacobsen and Schröger (2003) and Hsu et al. (2010), the data for the present study provided conclusive evidence for a larger difference between the classic and genuine MMN in the multi‐feature than in traditional auditory oddball paradigms. The considerably and consistently smaller *relative* contribution of the genuine MMN to the classic MMN in the multi‐feature oddball paradigm, compared to the traditional oddball paradigm, across all compared studies might possibly indicate compromised predictive processing in the multi‐feature paradigm presumably due to irregular context and modified transition probabilities. Replicating this finding in a single within‐subject design study with identical stimulus material appears feasible.

Furthermore, we observed a significant modulation of the amplitude difference between the classic and genuine MMN between features. That is, in the multi‐feature oddball paradigm the relative contribution of adaptation and physical difference is different between features, and one cannot conclude on genuine MMN amplitudes from classic MMN amplitudes (without a control condition). The genuine MMN in the multi‐feature oddball is not only distinctly smaller than the classic MMN but also considerably less reliable. Figure 2 shows the distributions of deviant minus standard and deviant minus control mean amplitudes in violin and box plots per feature. Only one out of 30 participants does not show a classic MMN for deviations in any feature. In contrast, only three quarters of the participants do show a genuine MMN in response to frequency, location, and duration deviants (adjusted time window) and only about half of the participants in response to intensity and duration deviants (canonical time window).

We did not observe a genuine MMN in response to intensity deviants. This lack of an intensity MMN must not be ascribed to low discriminability since participants were able to discriminate changes in intensity (d′ = 3.38). Moreover, the lack of a genuine intensity MMN also was not due to low power since Bayesian *t*‐tests rather showed strong evidence for the null model. As a genuine intensity MMN has been shown in the traditional oddball paradigm (incl. the equiprobable control condition; Jacobsen et al. 2003) this appears to be specific to the multi‐feature oddball paradigm and might possibly be due to the concurrent presentation of location and/or duration deviants, which makes intensity an unreliable cue. In the case of variable sound source locations, perceived constant intensity may result from softer or louder sounds presented from a closer or farther distance, respectively; conversely, variable perceived intensity may result from a constant‐loudness sound source at variable distance. Similarly, longer sounds are typically perceived as louder due to the integration of sound energy over time (up to about 200‐ms duration; Munson 1947; for review, see Scharf and Houtsma 1986). In the case of variable sound duration, perceived constant intensity may result from shorter, louder or longer, softer sounds, and conversely, variable perceived intensity may result from sounds that are constant in loudness but shorter or longer. In other words, the genuine MMN might be compromised in the multi‐feature oddball paradigm by the violation of the assumption of separated rather than integrated processing of sound features impairing the extraction of regularities. We did not observe a genuine duration MMN in the expected time range. This may be because the study by Pakarinen et al. (2007), which we used to define our canonical time windows, did not control for physical differences. These yield differences in the latencies of the exogenous components, which, in turn, may have contributed to the classic MMN (and shifted its apparent latency). We would like to note that the apparent peak latency difference between the classic and the genuine MMN observed in the traditional oddball study by Jacobsen and Schröger (2003) (10 ms) was considerably smaller than that observed in the present study despite their larger difference in sound duration (50 ms vs. 25 ms in the present study). We do not have a hypothesis why the genuine MMN observed in response to duration deviants did not reliably invert polarity at mastoidal sites even in an adjusted time window.

In sum, negative amplitudes in the deviant‐minus‐standard comparison in the auditory multi‐feature oddball paradigm do not permit conclusions on the presence or amplitude of the genuine MMN, which presumably reflects higher‐order processes beyond adaptation, such as genuine deviance detection, memory comparison, model updating, or predictive processing. Our results do not support the assertion that the MMN can be measured in the multi‐feature paradigm “without compromising the MMN amplitude” (Näätänen et al. 2004, 143); more precisely, it does not apply to the deviant minus control derived genuine MMN controlled for physical difference and adaptation.

### 4.2 The Need to Differentiate Adaptation From the Genuine MMN

In contexts where the assessment of the mere ability to discriminate deviants from standards during passive listening is relevant—without considering the underlying information processing enabling this discrimination—the multi‐feature oddball paradigm works well. However, it is necessary to consider the differential contributions of adaptation and predictive processing to the classic MMN observed in the multi‐feature oddball paradigm in any research questions or populations where these are relevant. This is, for example, the case in research on schizophrenia. The MMN is considered a potential biomarker of schizophrenia as MMN amplitudes have been found to be reduced in patients suffering from schizophrenia compared to healthy controls with high consistency (for review, see Michie et al. 2016; Schall 2016; Todd et al. 2012). The duration MMN was found to be slightly more sensitive than the frequency MMN (Umbricht and Krljes 2005). Glutamate NMDA receptor hypofunction has been considered a potential causal link between reduced MMN amplitudes and schizophrenia. The assumption of NMDA receptor hypofunction contributing to schizophrenia is well established, and pharmacological studies have shown that NMDA receptor impairment affects the MMN (Michie et al. 2016). It has been convincingly shown that NMDA is relevant in predictive processing; a neuronal model relying upon NMDA receptor synaptic transmission is sufficient for a predictive coding account of the MMN (Wacongne et al. 2012; Zhang et al. 2025). Importantly, Wacongne (2016) has also successfully demonstrated the effect of NMDA receptor dysfunction affecting the MMN but not adaptation in a neuronal model based on predictive coding. (Stimulus‐specific) adaptation potentially has a modulating function (Michie et al. 2016; Wacongne 2016) but reflects an NMDA‐independent process (Farley et al. 2010). Therefore, it appears essential to us to measure and understand the differential contributions of adaptation and predictive processing to the observed reduced MMN in schizophrenia. It might be particularly beneficial to include a control of adaptation (and physical difference) to the multi‐feature oddball paradigm as it is commonly applied in schizophrenia research in order to enhance specificity as well as sensitivity. We would like to note that this does also apply to most studies employing the traditional oddball paradigm in research on schizophrenia lacking a control of adaptation. This issue was discussed in detail by Todd et al. (2013).

In a more general sense, the application of the auditory multi‐feature oddball paradigm without control for adaptation and physical difference appears potentially problematic in any study examining higher order cortical contributions to deviance detection (e.g., predictive processes) where inference is drawn from differences in MMN amplitudes between features, conditions, or groups. Presumably this applies to many fields of MMN research, including psychiatry (e.g., using MMN feature profiles including duration and intensity, Fisher et al. 2008), music, speech and language including developmental aspects, or neural transmission speed (e.g., Kompus et al. 2015), and others. The differential contribution of context irregularity, transitional probabilities, feature integration, physical difference, and adaptation might considerably vary between features, conditions, and/or groups. Observed differences in the deviant minus standard derived classic MMN, thus, possibly may result from variable contributions of adaptation and genuine MMN. Similar effects may possibly be expected also in variations of the paradigm (e.g., language and music); this is, however, still to be empirically tested. These limitations may be lifted, at least partly, by the application of the suggested multi‐feature cascadic control paradigm still allowing the rapid measurement of four deviant features within 20–30 min (for 100–150 deviant and control trials). Additionally, due to possible effects of feature integration, we suggest avoiding the combination of location and intensity as well as intensity and duration sound features. Using sound features other than intensity and duration, for example, timbre or envelope, may show a more robust genuine MMN. The suggested cascadic control paradigm can be applied to any stimulus material that permits the parametric modulation of the manipulated features, such as speech (e.g., Pakarinen et al. 2009) or music (e.g., Vuust et al. 2016).

### 4.3 Conclusion

We found a substantial reduction of deviant minus control “genuine” MMN amplitudes compared to deviant minus standard “classic” MMN amplitudes in the multi‐feature oddball paradigm for frequency and location deviants; we did not find an MMN for intensity and presumably also duration deviants (at least not in the expected time window) when controlling for adaptation and physical difference in a multi‐feature cascadic control condition. The multi‐feature oddball paradigm including the suggested cascadic control condition allows the fast measurement of the genuine MMN for four deviant features controlled for adaptation and physical differences. We recommend including conditions controlling for adaptation and physical difference in the multi‐feature *as well as* in the traditional oddball paradigm in order to avoid overestimation of the genuine MMN presumably reflecting higher order cortical computations such as predictive processing and properly estimate the contribution of adaptation to the observed MMN.

## Author Contributions

**Andreas Widmann:** conceptualization, data curation, formal analysis, investigation, methodology, project administration, software, visualization, writing – original draft, writing – review and editing. **Erich Schröger:** conceptualization, writing – review and editing. **Nicole Wetzel:** conceptualization, funding acquisition, project administration, resources, writing – review and editing.

## Funding

This work was supported by the Deutsche Forschungsgemeinschaft (WE 5026/1‐2 and WE 5026/4‐1), the Leibniz Association (P58/2017), and the Center for Behavioral Brain Sciences (ZS/2016/04/78120).

## Conflicts of Interest

The authors declare no conflicts of interest.

## Data Availability

The aggregated data including the code used for data analysis are provided in the OSF repository (https://osf.io/adh3j/) in JASP format (and the corresponding html output). The raw data are available from the corresponding author upon request.

## Endnotes

^1^
A conference contribution by Vollmer et al. (2023) reported a “multi‐standard” control condition, but not an equiprobable control condition. Each feature level was presented in 33% of trials (in contrast to 8.3% in the multi‐feature oddball condition). Whereas physical identity was successfully controlled in their design, adaptation presumably contributed substantially to the observed deviant minus control ERPs.

^2^
We used intensity increment rather than decrement deviants (potentially resulting in enhanced ERP amplitudes) as this was required by the cascadic control condition. In the ERP analysis, we exclusively compare physically identical sounds. Therefore, we do not expect any undesirable side effects of using intensity increments rather than decrements.
